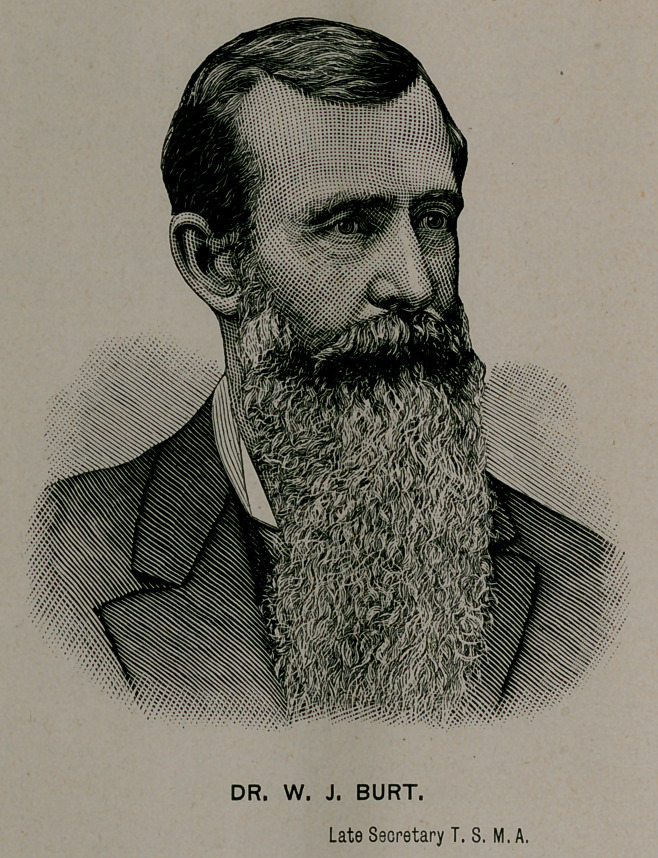# Dr. Wm. Jefferson Burt, Late Sec., T. S. M. L.

**Published:** 1887-04

**Authors:** 


### OBITUARY.

### DR. WILLIAM JEFFERSON BURT, LATE SECRETARY TEXAS STATE MEDICAL ASSOCIATION.

From Daniel's Texas Medical Journal for July, 1886.

The medical profession of Texas, and more particularly the State Medical Association sustained a sad, sad loss in the death of this estimable gentleman and physician. On the night of July 1st, 1886, he read an elaborate paper before the Travis County Medical Society. Having been complaining for several days previously of some bowel trouble, he took to bed that night and in one week and a day was no more. His death was calm and without a struggle. He passed away quietly at 4 a. m., Saturday, July 10th, and his remains were followed to the grave by the largest concourse of people that ever did honors to any deceased in Austin. During the impressive services at the church—by his confidential friend, companion and constant associate—the minister of the church of which he was a member—Rev. Dr. Smoot, strong men wept, and all classes, high and low, shared the great grief his untimely death brings on this community. He was universally beloved for his many excellent qualities ot head and heart.

Dr. Burt was born in Dawsonville, Ga., June 15, 1838. After finishing his literary education at Hiawasse and Mossy Creek colleges, east Tennessee, he studied medicine in 1858–9 in Georgia under Dr. John Hockenhull, and attended lectures in Atlanta, where he graduated in 1860.

In the same year he moved to Arkansas and entered the Confederate army as surgeon to the 17th Arkansas regiment. He continued in the service until the close of the war.

In 1865 he settled at Jacksonport, Ark., there continuing to practice his profession until the close of 1873, when he came to Austin, for his health, where he has resided ever since.

In 1879–80 he took a medical course in New York in the College of Physicians and Surgeons and in Bellevue Hospital Medical College. He was an active member of the Travis County Medical Association, of which at one time he was president. He was also a member of the American Medical Association.

At one time he was chairman of the board of managers of the Texas Insane Asylum and President of the Medical Examining Board of the sixteenth judicial district. During the years of 1884–85 he was city and county physician, and for six years Secretary of the Texas State Medical Association.

Dr. Burt was married in 1860 to Matilda Palmer, of Big Savannah, Ga. He leaves his wife and three sons—the oldest, Montrose Burt, being just 21, and the youngest 17 years of age.

He was one of the leading physicians of this county and city, and as such at times contributed some very interesting and valuable papers to the medical literature of the day. He was held in high esteem by all with whom he associated, and his death has caused a void which will be difficult to fill.

The entire medical profession of Austin attended his funeral, two members of the Travis County Medical Association acting as pall bearers, with two members from the Lodge of the Knights of Honor of which he was a member, and two representatives of his Church.

A meeting of the physicians of the city was held the day of his death, and Drs. W. A. Morris, R. M. Swearingen and F. E. Daniel were appointed a committee to draft resolutions expressive of the sentiments of the profession of the city on the death of their companion; and the following was adopted, ordered published in *The Statesman*, and a copy in manuscript sent to the family of the deceased.

"WHEREAS; He, 'without whose knowledge not a sparrow falleth to the ground,' has, in His inscrutable wisdom, removed from our midst our friend and fellow-physician, Dr. W. J. Burt, in the prime and vigor of his mature manhood, in mid-career of his usefulness as a citizen and a practitioner of the healing art, ere the measure of his years had been fulfilled;

*Resolved,* That it is with the sincerest regret and sorrow that we are deprived thus prematurely of his companionship and services, and that we will ever cherish for his memory the kindliest sentiments.

*Resolved,* That in the death of Dr. Burt the medical profession at large has sustained an immeasurable loss; in its organized capacity, one of its most useful, active, zealous and progressive workers; an officer whose labor and talents have contributed very largely to the success and progress of the Association; that the city of Austin has been bereft of one of her most liberal and enlightened, and really useful citizens; and that in every relation of life, his was an example worthy of emulation; a kind, loving and provident husband and father, a devoted Christian worker in the Church, a useful and liberal-minded citizen, a staunch friend, and an enlightened and benevolent physician. And as we consign to earth the mortal remains of our cherished friend and fellow-worker in a cause to which he lent lustre, and adorned, we experience the deepest grief and sorrow. The thought is agonizing, that no more in this life will we meet his genial smile, his cordial and honest grasp, nor in the sick room hear his sympathetic and encouraging voice, nor listen to the words of wisdom and learning that were wont to delight us in our society halls; yet in all humiliation and reverence we do humbly bow to the will of the Great Physician, and console us that He, too, is the Great Healer of our hearts, while we say 'Thy will, not mine, be done, Oh God!'

Peace to thy ashes, Oh genial, true-hearted Christian friend. May the Tender Shepherd comfort, console and support thy little flock, loved so well, in the bitterness of their grief, in this, the sad hour of their affliction and heart desolation."

## Figures and Tables

**Figure f1:**